# Orbital masses: a review of CT imaging characteristics

**DOI:** 10.3389/fopht.2025.1685141

**Published:** 2025-11-14

**Authors:** Eran Levanon, Gahl Greenberg, Yael Lustig-Barzelay, Daphna Landau-Prat, Guy J. Ben Simon

**Affiliations:** 1 Orbital Ophthalmic Plastic and Lacrimal Surgery Institute, Sheba Medical Center, Ramat Gan, Israel; 2 Diagnostic Imaging Department, Neuroradiology Unit, Sheba Medical Center, Ramat Gan, Israel

**Keywords:** computed tomography, orbital masses, vascular malformation, lacrimal gland tumor, dermoid cyst, pleomorphic adenoma, myositis, imaging features

## Abstract

Orbital masses include a diverse spectrum of benign, malignant, inflammatory, and vascular lesions in pediatric and adult patients. Accurately diagnosing the type of lesion is critical, as management strategies differ significantly. Advanced imaging is therefore essential, and computed tomography (CT) is central to orbital evaluation. We reviewed the literature to synthesize evidence on CT features across common orbital pathologies and correlated imaging with clinical presentation to emphasize diagnostic relevance. CT characteristics are summarized for vascular lesions (cavernous venous malformation, lymphatic malformation), inflammatory conditions (orbital myositis, dacryoadenitis), benign lesions (dermoid cyst, pleomorphic adenoma), and malignant lesions (lacrimal gland lymphoma, adenoid cystic carcinoma, rhabdomyosarcoma). We present characteristic patterns of location, morphology, enhancement, and bone change, with practical discriminators and common pitfalls to aid differentiation. When used alongside clinical context, CT remains a preferred modality in many clinical settings due to its rapid acquisition, wide availability, and reliable depiction of bone and calcifications. It supports accurate diagnosis and informed management decisions in time-critical settings. This review provides a structured reference for interpreting CT findings across a wide range of orbital disease.

## Introduction

Orbital masses in pediatric and adult populations include a wide range of conditions—benign tumors, malignant neoplasms, and inflammatory conditions. Accurately diagnosing the type of lesion is essential, as management strategies differ significantly, ranging from conservative monitoring to aggressive surgical or systemic therapy. Clinical presentation alone is often insufficient for precise diagnosis, reinforcing the vital role of advanced imaging in guiding treatment decisions (1, 2).

Benign lesions, such as cavernous venous malformations, dermoid cysts, and pleomorphic adenomas, typically present with slow progression and well-defined margins and therefore will often cause smooth, non-destructive, bone remodeling. Inflammatory conditions, including orbital myositis and dacryoadenitis, may mimic neoplasia, exhibiting muscle enlargement or glandular swelling with variable contrast enhancement and adjacent fat stranding, but these conditions usually respond to medical therapy (3). In contrast, malignant tumors such as rhabdomyosarcoma, lymphoma, and adenoid cystic carcinoma often show rapid growth, infiltrative borders, bone erosion, and aggressive enhancement patterns on imaging studies. Early identification of these characteristics is critical for timely intervention (4, 5).

Periodic advances in multidetector CT have improved the evaluation of orbital masses by enhancing lesion localization as well as bony/tissue interface characterization, thus aiding in lesion assessment and staging. The essence of CT remains an image of densities based on a well-known scale (Hounsfield units—HU), which represents a standardized, quantitative measurement of radiodensity in which water equals 0, air is approximately −1,000, and bone or metals are 1,000 or higher; hemorrhage is usually within +40 to +80, whereas fat falls within the range of -50 to -150. The recent introduction of photon counting CT (PCCT) shows great promise, enabling ultra-high spatial resolution with superb bone detail and improved CT angiography features with visualization of small-sized vessels, previously undetectable. PCCT also allows spectral imaging (i.e., iodine maps) and improved metal artifact reduction. CT remains the modality of choice in many clinical settings due to its speed, accessibility, and ability to visualize bone, calcifications, and lesion enhancement. While MRI offers superior tissue characteristics, CT is still preferred in scenarios of acute trauma, 3D surgical planning, and urgent diagnostic settings. Emerging AI tools are beginning to support CT-based differentiation between benign and malignant lesions, further expanding its diagnostic value in orbital imaging (6–8).

This review systematically examines the CT features of common orbital pathologies across pediatric and adult populations. By correlating imaging findings with clinical presentation, we aim to enhance differential diagnosis, optimize patient management, and support evidence-based decision-making in orbital disease. CT is one component of multimodal orbital imaging, and additional imaging is often required to fully characterize lesions and plan management. Within this framework, we focus on the indications and uses of CT.

## Vascular lesions

### Cavernous venous malformation

#### Clinical overview

Cavernous venous malformation (CVM), previously termed cavernous hemangioma, is the most common benign orbital mass in adults. It typically affects individuals in their fourth to fifth decade of life, with a noted female predominance. Clinically, CVMs present with painless axial proptosis, often progressing slowly over months or years. Visual impairment may result from optic nerve compression, particularly in apical lesions. Gaze-evoked amaurosis and diplopia are less common symptoms observed in both intraconal and extraconal orbital masses, likely due to transient axonal conduction block or optic nerve ischemia during eye movement. Although generally solitary and unilateral, bilateral cases have been reported. CVMs are well-encapsulated, noninfiltrative lesions composed of large, blood-filled vascular spaces lined by mature endothelium, supported by fibrous stroma. Their slow growth and lack of endothelial proliferation classify them as low-flow venous malformations rather than true neoplasms (9–11).

#### CT imaging features

**Figure 1:**
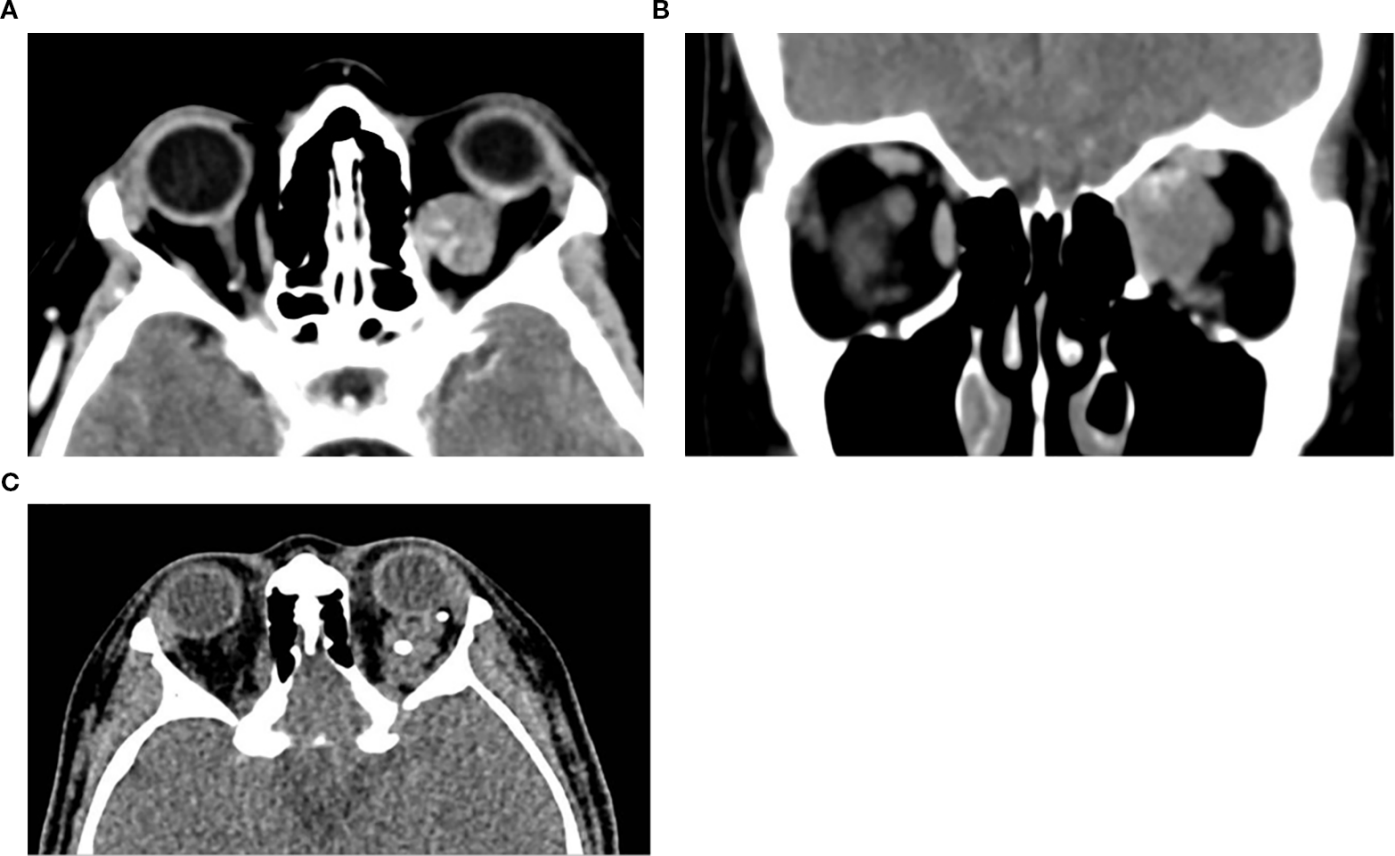
Axial **(a)** and coronal **(b)** post-contrast CT image of the orbits in a 65-year-old female patient demonstrates a well-defined, circumscribed, intraconal lesion in the left orbit located between the medial rectus muscle and the optic nerve, with associated proptosis and mild globe indentation. The lesion exhibits early focal stippled enhancement following contrast administration. These imaging features are characteristic of a CVM. **(c)** Axial non-contrast CT of a 16-year-old female patient with left orbital venous malformation, showing multiple well-circumscribed hyperdense round foci within the intraconal lesion, consistent with phleboliths, a typical finding in venous malformations.

CT imaging plays an important role in diagnosing and planning the surgical management of CVMs. On non-contrast CT, CVMs typically appear as well-circumscribed, fairly homogeneous, round or ovoid soft-tissue masses. They may be found in any compartment, but the majority, roughly 80%, are located within the intraconal space, usually lateral to the optic nerve. They are isoattenuating or slightly hyperattenuating relative to extraocular muscles. A hallmark feature of CVM is delayed progressive enhancement: initial focal peripheral or central enhancement which gradually progresses to incomplete or heterogeneous filling in delayed phases. This “centripetal” enhancement pattern reflects their slow-flow vascular channels and helps differentiate CVMs from other orbital tumors (see **Figure 1**) such as schwannomas or lymphomas. In 88.5% of cases, delayed incomplete filling is noted (9, 12).

Bone remodeling may be seen in larger lesions. Due to its fast acquisition, CT is particularly useful for identifying the early focal enhancement sign, which may be missed on delayed scans, CT is also useful for surgical planning by delineating lesion borders and proximity to critical structures (3, 10).

### Lymphatic malformation

#### Clinical overview

Lymphatic malformations are congenital vascular anomalies characterized by abnormally formed lymphatic channels, typically diagnosed in childhood. They commonly present in the first decade of life, often with slowly progressive or intermittent proptosis, globe displacement, and pain due to spontaneous intralesional hemorrhage or infection. These lesions can expand rapidly during upper respiratory tract infections or trauma, producing acute symptoms such as vision loss or periocular swelling (13). Although benign, their unpredictable growth, trans-spatial extension, and potential for functional impairment necessitate careful monitoring and timely intervention. Histologically, these lesions consist of dilated lymphatic vessels, often interspersed with venous elements, and may demonstrate reactive lymphoid aggregates (14, 15). Lymphatic malformations can be macrocystic, microcystic, or mixed lesions based on imaging characteristics. A diameter of 1 to 2cm has been used as a cutoff between macrocystic and microcystic designations, while an alternative practical definition is whether a cyst is amenable to aspiration (16, 17).

#### CT imaging features

**Figure 2:**
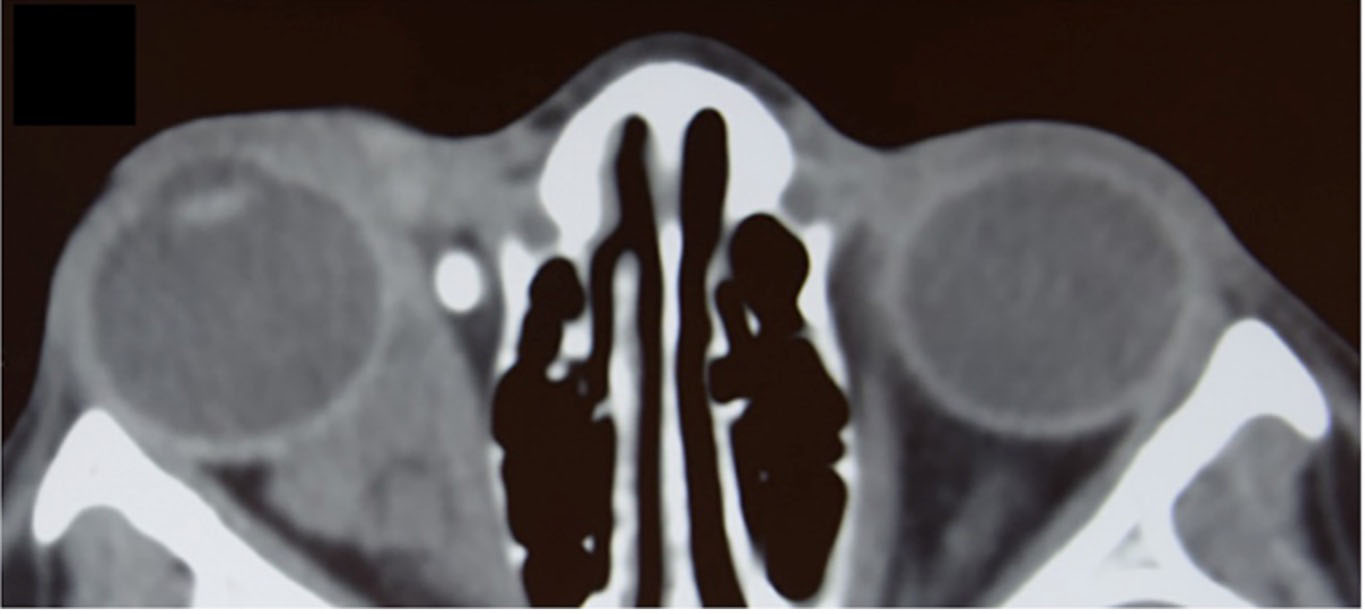
Axial non-contrast CT of the orbits in a 12-year-old girl demonstrating a lobulated, mostly iso-attenuating mass in the right orbit with both intraconal and extraconal components, including nasal preseptal extension. The lesion is causing mild globe displacement without bony erosion. A phlebolith is noted in the anteromedial orbit, consistent with a slow-flow vascular lesion and supporting a venous component within the overall lesion. Therefore, the lesion is classified as a lymphatico-venous malformation. Diagnosis was reached by further assessment and imaging.

Lymphatic malformations typically appear as lobulated, poorly circumscribed, localized, or trans-spatial lesions involving both intraconal and extraconal compartments. The density on non-contrast CT is heterogeneous, reflecting cystic components and fluid–fluid levels, particularly in cases with prior hemorrhage. These fluid–fluid levels are highly suggestive of the diagnosis (1, 14). Calcification is rare, and adjacent bone remodeling is typically absent. Contrast enhancement, when present, is usually peripheral or septal, corresponding to enhancing cyst walls or internal septations (13). The absence of solid nodular enhancement helps differentiate lymphatic malformations from vascular tumors such as hemangiomas. Importantly, the lesion’s appearance may vary over time depending on the state of hemorrhage or infection, and interval enlargement is common during acute episodes. For suspected vascular lesions, MRI is the primary modality to delineate extent and flow characteristics, with ultrasound as a useful adjunct. CT is essential to evaluate bony anatomy and orbital compartment involvement and identify acute hemorrhagic components. This assists in treatment planning, especially when considering surgical debulking or sclerotherapy, which remains the mainstay of therapy for symptomatic lesions (1, 14, 15) (**Figure 2**).

## Inflammatory lesions

### Orbital myositis

#### Clinical overview

Orbital myositis is an inflammatory condition primarily involving the extraocular muscles, often idiopathic in origin and classified within idiopathic orbital inflammation syndromes (IOIS). It presents predominantly in young to middle-aged adults, with peak incidence between the third and fourth decades. Clinically, patients typically exhibit acute to subacute onset of painful ophthalmoplegia accompanied by orbital pain exacerbated by ocular movements, diplopia, eyelid swelling, and conjunctival injection. Although classically unilateral, bilateral involvement may occur in recurrent or chronic forms. The medial and lateral recti are most frequently affected, followed by the superior and inferior recti, and rarely the oblique muscles. A migratory pattern has been described in orbital myositis, with recurrences affecting different extraoculuar muscles in either the same or contralateral orbit. The disease is notable for its prompt responsiveness to corticosteroids; however, the recurrence rates are significant, necessitating long-term management and sometimes steroid-sparing therapies to avoid complications from chronic steroid use (18–21).

#### CT imaging features

**Figure 3:**
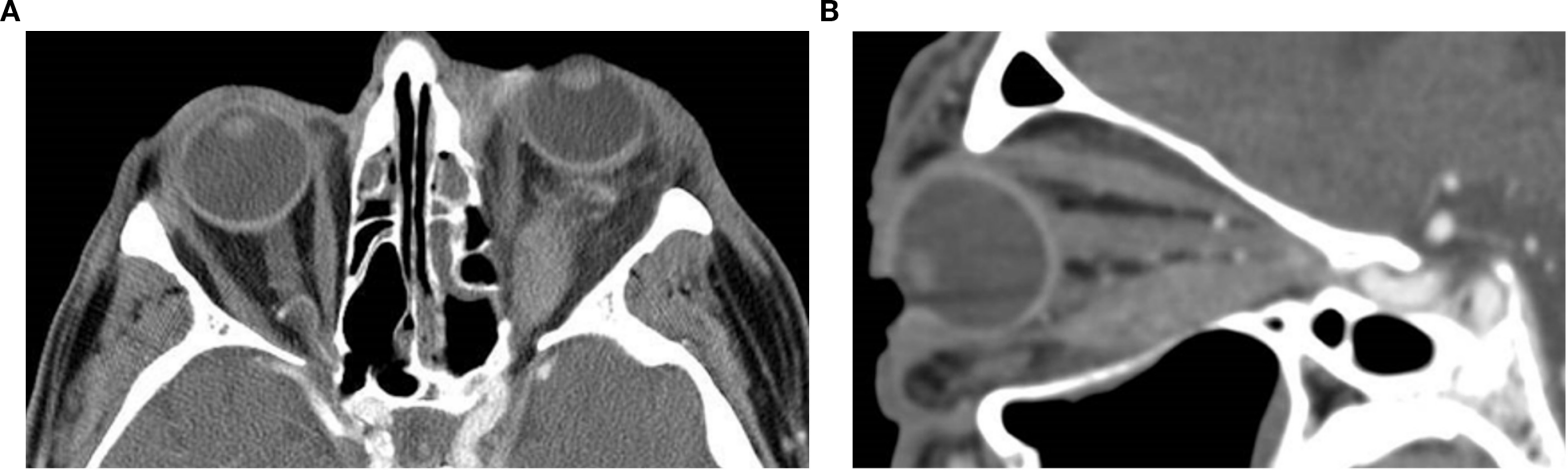
Axial **(a)** and sagittal oblique reformat **(b)** post-contrast CT images of the orbits in a 55-year-old male patient with left-sided orbital myositis demonstrate diffuse thickening and enhancement of the inferior rectus muscle with associated moderate axial proptosis. There is associated infiltration of the intraconal and extraconal fat, particularly in the temporal quadrant. The remaining extraocular muscles are unremarkable. The muscle enlargement results in mild medial displacement of the optic nerve complex (not shown), without significant retrobulbar crowding or orbital apex involvement.

On CT, idiopathic orbital myositis typically shows fusiform enlargement of the affected extraocular muscle. Although tendon involvement is often mentioned, published series report tendon thickening in fewer than half of cases, so tendon sparing does not exclude the diagnosis (approximately 41%–47%) (22–24). In thyroid-associated orbitopathy, the tendon is usually spared, yet limited tendon enlargement has been reported in a small minority of cases. Taken together, tendon appearance should be interpreted in context and not used in isolation for definitive differentiation (25). Muscle enlargement generally measures between 6 and 10mm in diameter, with sharp margins evident in acute cases (18), whereas chronic presentations may exhibit less-defined borders and associated inflammatory changes in adjacent orbital fat (this can occur in acute settings as well). Following contrast administration, pronounced enhancement of affected muscles is typical. Unlike malignancies or metastases, orbital myositis rarely shows nodular or focal lesions, calcifications, hemorrhage, or bone erosion; such findings necessitate further investigation to exclude other etiologies. CT may be the first modality to provide sufficient imaging clues for orbital myositis, aiding in accurate diagnosis and therapeutic planning and, in certain circumstances, may be used for monitoring of treatment response. However, MRI is often preferred due to its superior soft tissue contrast, ability to detect muscle edema, and improved distinction between active inflammation and chronic changes (18, 19, 26, 27). In practice, cases with this appearance should have clinical reassessment and repeat imaging to confirm resolution, and a lack of improvement should prompt further evaluation to exclude neoplastic etiologies (**Figure 3**).

### Dacryoadenitis

#### Clinical overview

Dacryoadenitis is an inflammatory condition of the lacrimal gland that may be infectious, autoimmune, or idiopathic in etiology. The bacterial form, although rare, is more frequently observed in adults than in children and may progress to suppurative inflammation with abscess formation. Acute bacterial dacryoadenitis typically presents with pain, eyelid swelling, and erythema in the superolateral orbit, often accompanied by mechanical ptosis, globe displacement, and extraocular motility restriction. Methicillin-sensitive and methicillin-resistant *Staphylococcus aureus* is the most commonly implicated pathogen, with an increasing prevalence of community-acquired MRSA noted in recent years (28, 29). Prompt recognition and early imaging are essential to guide management and prevent sequelae such as compressive optic neuropathy (29–31).

#### CT imaging features

**Figure 4:**
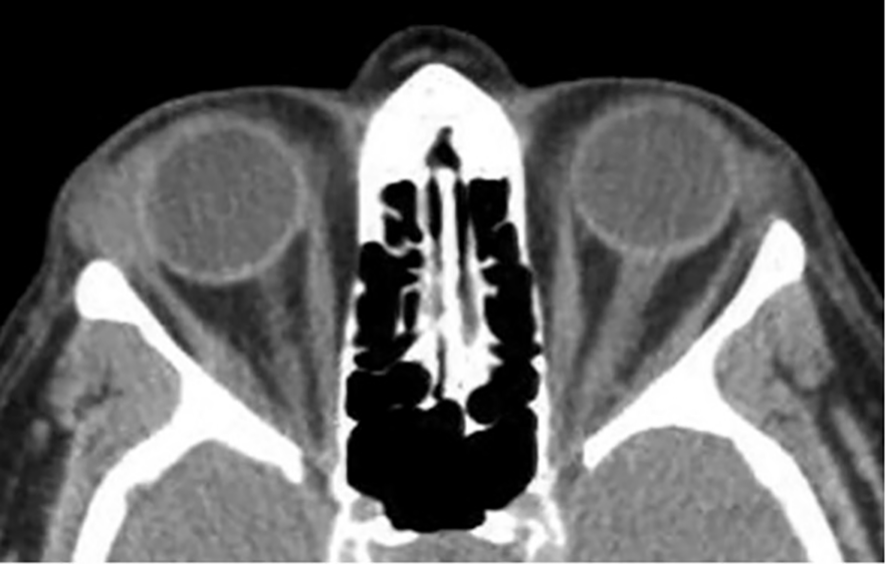
Axial non-contrast CT image of the orbits in a 71-year-old female patient showing diffuse enlargement of the right lacrimal gland with relatively homogeneous soft tissue attenuation and mild adjacent preseptal fat stranding, a hazy, poorly defined increase in fat attenuation that reflects edema or inflammation. The findings are compatible with dacryoadenitis. The diagnosis was confirmed on biopsy.

CT is the adequate imaging modality of choice in the urgent evaluation of suspected bacterial dacryoadenitis, particularly to assess for abscess formation. Typical CT findings include enlargement and enhancement of the lacrimal gland, which may be accompanied by a fluid-filled, rim-enhancing hypodense collection consistent with abscess. Surrounding inflammatory changes such as preseptal and postseptal soft tissue swelling, fat stranding in the superolateral orbit, and globe displacement may be observed (28, 29). In rare cases, adjacent sinus opacification, particularly in the ethmoid sinus, supports the diagnosis and implicates a possible source of contiguous infection. In this context, the lacrimal gland, when affected, is typically involved secondarily as part of the diffuse orbital. Bone involvement, though uncommon, may include erosion of the orbital wall in advanced cases (29, 30, 32) (**Figure 4**).

## Benign neoplasms

### Dermoid/epidermoid cysts

#### Clinical overview

Dermoid cysts are the most common orbital cystic lesions in children, accounting for up to 46% of orbital masses in this population (1). They arise from ectodermal sequestration along embryonic suture lines, typically during fetal development, and contain dermal appendages such as hair follicles, sebaceous glands, and keratinized epithelium (13). Most are diagnosed within the first decade of life, often presenting in infancy or early childhood. Clinically, superficial dermoids appear as painless, slowly enlarging masses along the lateral brow, whereas deeper orbital cysts may cause gradual proptosis or globe displacement without significant inflammation. Though benign, rupture or secondary infection may trigger acute inflammation. The frontozygomatic suture is the most common site of origin for periorbital dermoids, and although rare, deeper orbital lesions can be misdiagnosed due to their nonspecific presentation (33). Epidermoid cysts are another common type of orbital cystic lesion, which tend to present later in life compared to dermoid cysts. Histopathologically, both dermoid and epidermoid cysts are lined by keratinized stratified squamous epithelium. However, dermoid cysts contain dermal adnexal structures such as hair follicles, sebaceous glands, and sweat glands, whereas epidermoid cysts do not (34, 35).

#### CT imaging features

**Figure 5:**
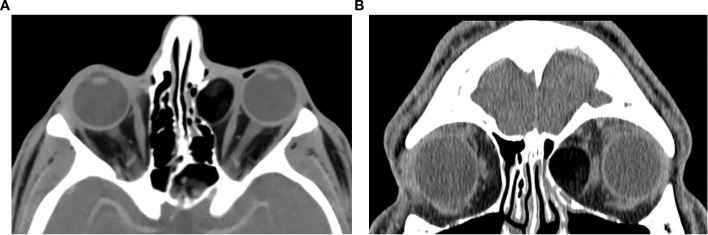
Axial **(a)** and coronal **(b)** post-contrast CT images of the orbits in a 26-year-old male patient reveal a well-defined, mostly fatty, extraconal lesion located nasally, medial to the medial rectus muscle in the left orbit. The lesion demonstrates predominantly fat attenuation (approximately –105 HU), with slightly denser internal components and peripheral thin rim enhancement. In this location, the appearance favors a dermoid cyst, whose lipid-rich contents produce fat attenuation, overall consistent with a benign cystic lesion. The mass exerts a moderate mass effect on the left medial rectus muscle with slight globe displacement and causes thinning and lateral bowing of the adjacent left lamina papyracea, suggestive of a chronic benign process. The diagnosis was confirmed on excision.

These lesions are typically located in the superotemporal orbit, commonly extraconal and anterior. On non-contrast CT, dermoid cysts appear as well-circumscribed, low attenuation lesions consistent with fat density, −100 to −50 HU (see “Introduction”). They may show internal fluid–fluid levels, soft tissue components, or calcifications depending on their content. Bony remodeling or scalloping of the adjacent orbital wall is frequent, particularly in long-standing lesions, and may be smooth or expansile (1, 36, 37). The presence of fat attenuation and adjacent bone remodeling is highly suggestive of a dermoid and aids in differentiating it from other cystic lesions such as epidermoid cysts or solid pediatric orbital masses (13, 35). Unlike malignant masses, dermoid cysts lack aggressive enhancement patterns and rarely infiltrate surrounding tissues. Contrast-enhanced CT may show thin rim enhancement if the cyst is inflamed or ruptured. Accurate delineation of lesion margins and assessment of adjacent bone are crucial for surgical planning, particularly to avoid rupture and minimize recurrence risk during excision (37) (**Figure 5**).

### Pleomorphic adenoma

#### Clinical overview

Pleomorphic adenoma of the lacrimal gland is the most common benign epithelial tumor of this structure, typically originating from the orbital lobe. It primarily affects adults in the fourth to the sixth decade of life. The classic presentation is an insidiously progressive, painless mass in the superolateral orbit, often accompanied by ptosis, proptosis, or globe displacement. However, atypical features such as inflammation, pain, or subcutaneous nodules may mimic orbital cellulitis or malignancy, particularly in recurrent or long-standing lesions (38). Tumor recurrence is commonly linked to prior incomplete excision or capsular violation and carries a risk of multifocal spread or malignant transformation over time (39). Long-term follow-up is therefore essential, especially as recurrence may occur decades after the initial surgery. Complete *en bloc* resection without prior biopsy remains the gold standard to minimize recurrence and prevent malignant degeneration (40, 41).

#### CT imaging features

**Figure 6:**
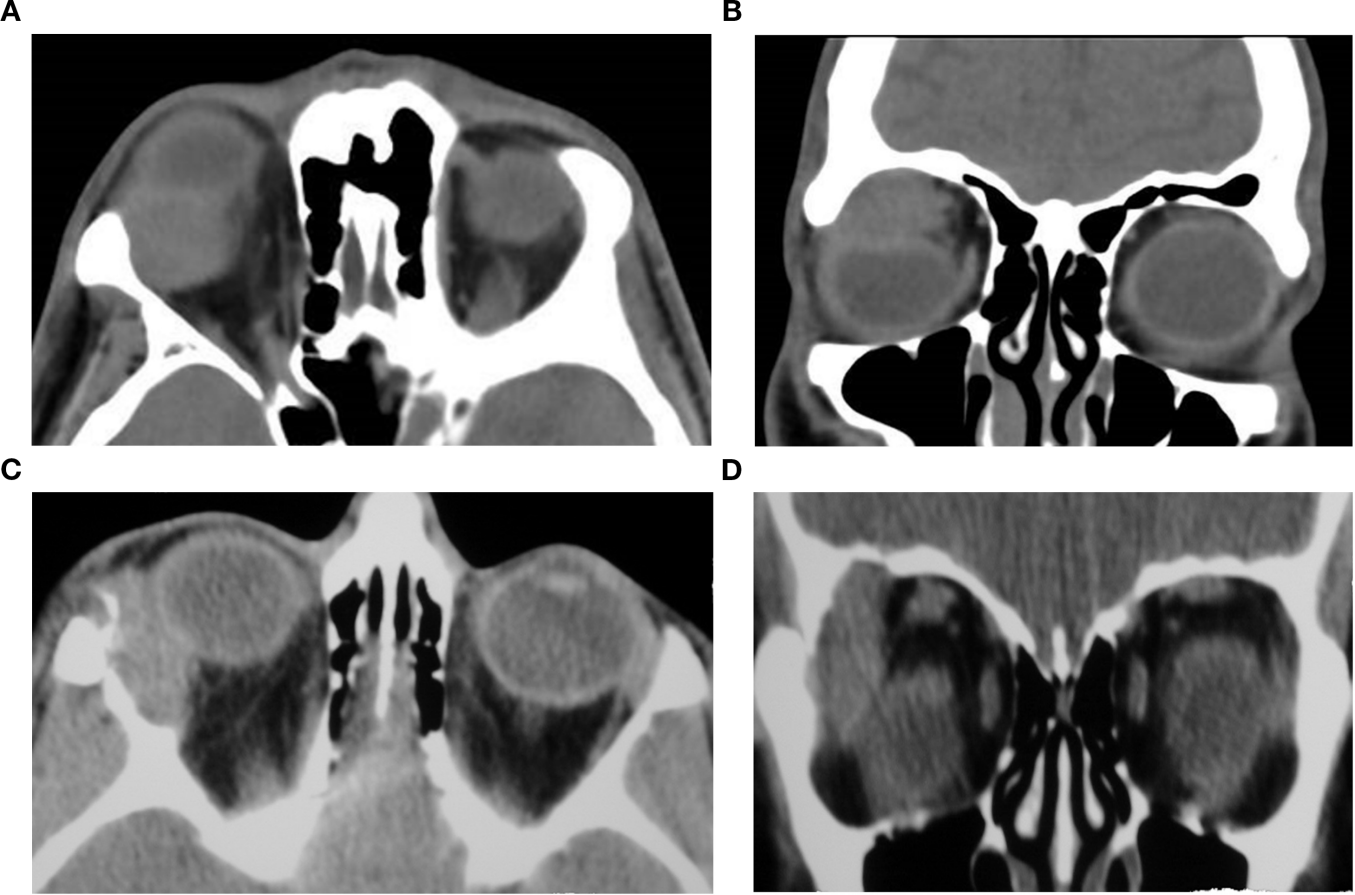
Axial **(A)** and coronal **(B)** CT images of a 37-year-old male patient demonstrating a space-occupying soft-tissue lesion centered in the lacrimal fossa of the right orbit, causing smooth remodeling of the superolateral orbital roof as seen on **(B)**, consistent with pleomorphic adenoma of the lacrimal gland. The lesion results in hypoglobus and proptosis, with moderate indentation of the superior scleral contour. Axial **(C)** and coronal **(D)** non-contrast CT images of a 45-year-old female with recurrent pleomorphic adenoma of the right lacrimal gland. **(C)** shows a partially circumscribed, lobulated soft-tissue mass in the lacrimal fossa causing inferomedial globe displacement; **(D)** confirms the superolateral location of the lesion within the orbit. Both images show bony changes from prior lateral orbitotomy. Histopathology after the initial removal attempt confirmed pleomorphic adenoma.

Pleomorphic adenoma of the gland typically appears as a well-circumscribed, smoothly marginated extraconal mass situated in the lacrimal fossa. The lesion is usually isodense to extraocular muscles on non-contrast CT and exhibits mild to moderate, often heterogeneous, enhancement following contrast administration (40, 42). Primary tumors tend to present with homogeneous architecture and may remodel adjacent bone in a shallow, smooth fashion. In contrast, recurrent tumors often display a lobulated or multinodular morphology, internal heterogeneity, and adjacent bone erosion or scalloping, which may mimic malignancy despite benign histology (39). Rare cases show calcification or atypical inflammatory signs, as seen in acute presentations (38). Features on CT that would favor malignancy include ill-defined margins, internal heterogeneity, calcifications, and bony invasion (43). Recurrent pleomorphic adenoma tends to develop multifocally and may be widespread in the previous operative field. On CT, recurrent tumor nodules are often associated with irregular bony erosion and remodeling despite these recurrences being usually benign. Repeated recurrence may require further surgery, posing a lifelong risk of significant morbidity and a potential for malignant transformation (39) (**Figures 6a–d**).

**Figure 6c** (axial) and **Figure 6d** (coronal) present non-contrast CT images of a 45 year-old-female with recurrent pleomorphic adenoma of the lacrimal gland in the right orbit. (**Figure 6c**) Axial section demonstrates a partially-circumscribed, lobulated soft-tissue mass in the lacrimal fossa causing inferomedial displacement of the globe. (**Figure 6d**) Coronal section confirms the supero-lateral location of the lesion within the orbit. Both images portray the bony changes following previous lateral orbitotomy. Biopsy ascertained after initial removal attempt.

## Malignant neoplasms

### Lacrimal gland lymphoma

#### Clinical overview

Lacrimal gland lymphoma is a malignant lymphoproliferative disorder, most commonly classified as extranodal marginal zone B-cell lymphoma of mucosa-associated lymphoid tissue (MALT). This section highlights lacrimal presentations, yet most orbital lymphomas are outside the lacrimal gland. Lacrimal cases comprise approximately 7% to 26% of ocular adnexal lymphomas, and the CT features apply across compartments (44). It primarily affects older adults, with a peak incidence in the sixth to the seventh decade of life. Clinically, it often presents as a painless, slowly progressive superolateral orbital mass, typically unilateral, and associated with ptosis, proptosis, or displacement of the globe. Systemic symptoms are rare, but patients may have a history of chronic autoimmune diseases such as Sjögren syndrome or rheumatoid arthritis, which can predispose to MALT lymphoma through chronic antigenic stimulation (45). Orbital MALT lymphoma is among the most common primary orbital malignancies, with a generally indolent course but a risk for systemic spread, emphasizing the importance of early diagnosis and biopsy. While CT may be useful to assess bony involvement, MRI remains the preferred modality for lacrimal gland evaluation due to its superior soft tissue contrast. Restricted diffusion on MRI, specifically, serves as a potentially viable tool in differentiating lymphoma from inflammatory lesions (46).

#### CT imaging features

**Figure 7:**
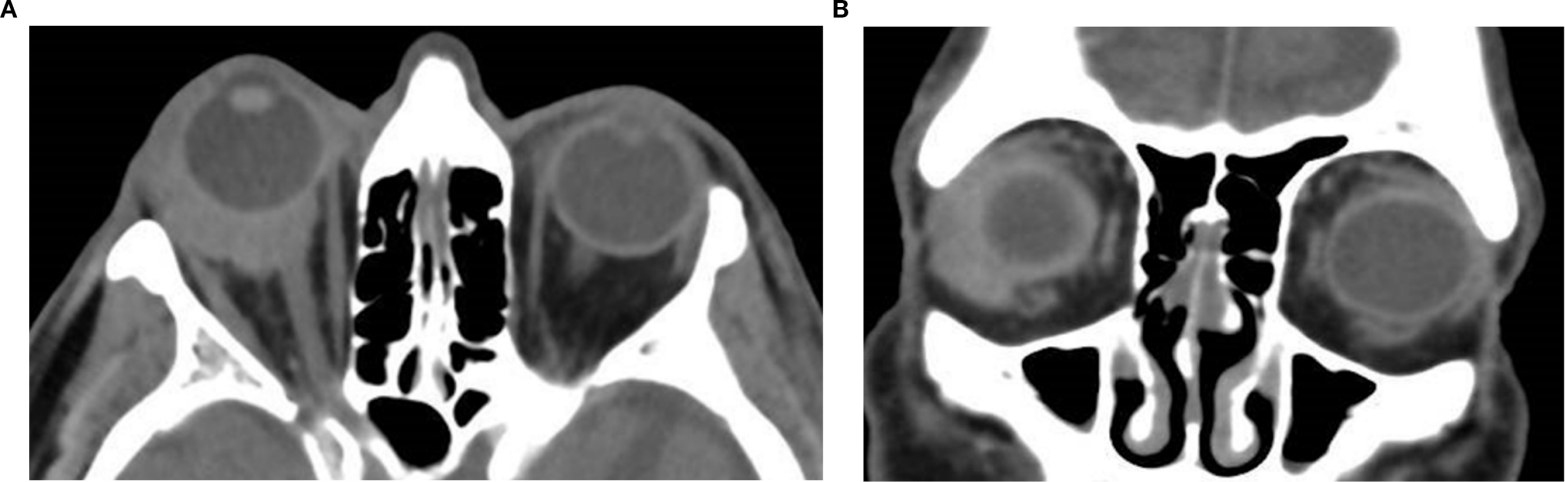
Axial **(a)** and coronal **(b)** non-contrast CT image of a 74-year-old male patient showing a homogeneous, well-defined soft tissue mass in the right lacrimal gland fossa. The lesion is hyperdense relative to orbital fat, demonstrates molding to adjacent structures with associated proptosis, and lacks bone erosion; biopsy confirmed it as lacrimal gland lymphoma.

On CT, lacrimal gland lymphoma typically appears as a relatively homogeneous, extraconal mass centered in the lacrimal fossa, frequently molded around the globe. Non-contrast images show an iso- to hyperdense lesion relative to muscle, while post-contrast scans demonstrate uniform enhancement. A notable imaging feature to search for in cases of lymphoma is the “wedge sign,” which is considered present when the lacrimal gland extends posteriorly beyond the anterior edge of the trigone of the greater wing of the sphenoid, between the lateral rectus muscle and the lateral orbital wall (47). Although more frequently associated with carcinoma, the wedge sign’s presence in lymphoma signifies aggressive behavior and may help differentiate it from benign inflammatory conditions such as dacryoadenitis (48). Unlike carcinomas, lymphomas rarely show calcification or bone remodeling (**Figure 7**).

It is worth mentioning that chronic inflammation arising from autoimmune conditions (particularly in Sjögren syndrome) may lead to cyst formation within the gland, with associated MALT lymphoma (45).

### Adenoid cystic carcinoma

#### Clinical overview

Adenoid cystic carcinoma (ACC) is the most common malignant epithelial tumor of the lacrimal gland, typically originating from the orbital lobe. It usually affects adults in the fourth to the sixth decade of life but has been reported in younger individuals as well. Clinically, ACC presents with a progressively enlarging mass associated with pain due to perineural invasion, proptosis, and sometimes diplopia or globe displacement. Histologically, it demonstrates cribriform, tubular, or solid growth patterns, with the solid subtype associated with the worst prognosis (49). Despite its slow growth rate, the tumor is highly invasive, prone to perineural spread, and demonstrates a high recurrence rate even after surgical resection and radiotherapy. Rarely, it may arise from ectopic lacrimal gland tissue in atypical orbital locations, such as the superonasal orbit (50, 51).

#### CT imaging features

**Figure 8:**
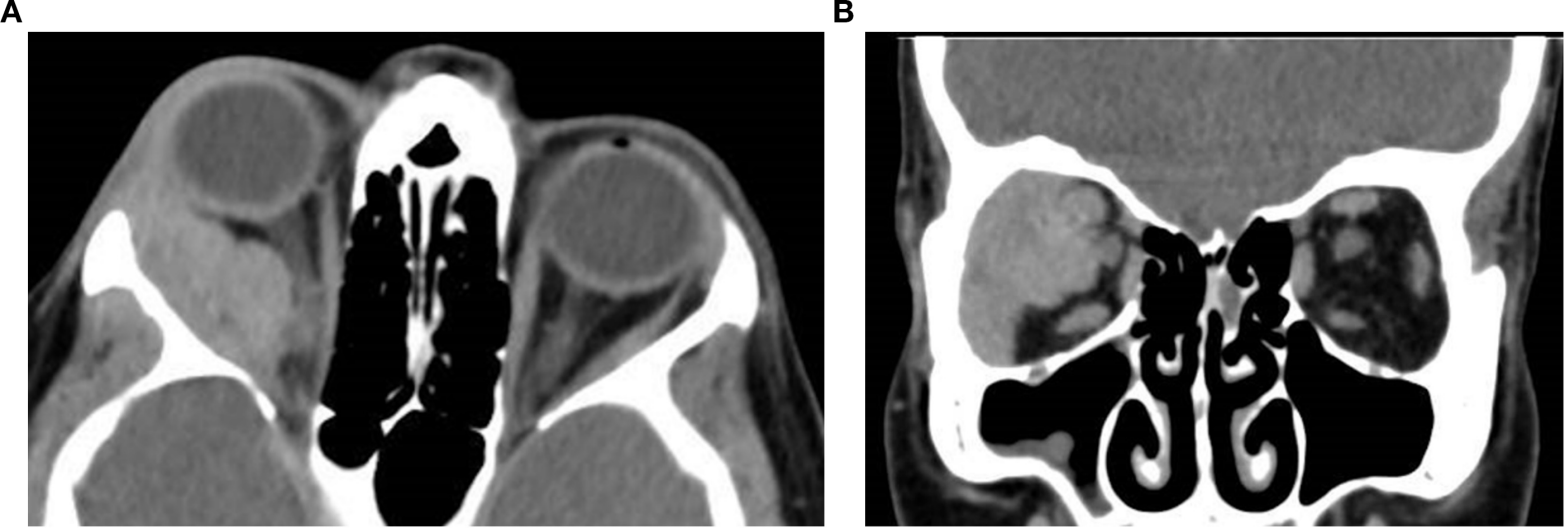
Axial **(a)** and coronal **(b)** post-contrast CT images of a 45-year-old female patient with adenoid cystic carcinoma of the right lacrimal gland. The images demonstrate an ill-defined, infiltrative soft tissue mass centered in the superolateral orbit, involving the lacrimal gland and extending into the extraconal space, with apparent displacement of the globe and the optic nerve. Subtle contour irregularity of the lateral orbital wall and zygoma was noted, with extension into the infratemporal fossa.

ACC commonly appears as an extraconal, irregular, soft tissue mass in the superolateral orbit. The margins may be ill-defined, and the lesion often shows bone erosion or destruction, which are hallmarks of its aggressive nature (52). However, rare cases may mimic benign tumors by demonstrating smooth contours and adjacent bone remodeling rather than destruction. Non-contrast CT typically shows an iso- to slightly hypodense mass relative to muscle, and contrast-enhanced CT reveals moderate to marked heterogeneous enhancement. In large lesions, heterogeneous density and cystic areas may be present. ACC rarely demonstrates calcifications. Perineural invasion, frequent in ACC, can extend along the ophthalmic division of the trigeminal nerve. Although CT is limited in detecting early perineural spread, it may eventually show the widening of the bony neural canal. Tumors can extend posteriorly, sometimes presenting as a “wedge sign” as mentioned previously, which refers to a triangular soft tissue extension between the lateral rectus and lateral orbital wall or between the superior rectus and the orbital roof. This finding is more commonly associated with aggressive lacrimal gland malignancies, such as ACC. Recognition of these features, particularly bone destruction and heterogeneous enhancement, is essential to distinguish ACC from benign lacrimal tumors such as pleomorphic adenoma (49) (**Figure 8**).

### Rhabdomyosarcoma

#### Clinical overview

Rhabdomyosarcoma (RMS) is the most common primary malignant orbital tumor of childhood, accounting for approximately 10% of all RMS cases. It arises from undifferentiated mesenchymal cells capable of skeletal muscle differentiation. The embryonal subtype predominates in orbital cases, comprising approximately 84% of tumors, with alveolar, spindle cell/sclerosing, and pleomorphic forms being less common but associated with a poorer prognosis (53). Orbital RMS typically presents in the first decade of life, with a median age of 7 to 8 years and a slight male predominance. Clinically, it manifests as rapidly progressive, unilateral proptosis, often accompanied by globe displacement, eyelid swelling, chemosis, or pain. Globe displacement typically occurs in a direction opposite to the tumor’s location and may be accompanied by distortion of the globe’s shape, although direct invasion of the globe is rare. Although RMS can mimic inflammatory or vascular lesions, its aggressive nature necessitates urgent diagnosis and treatment. Prompt initiation of combined chemotherapy and radiotherapy, preceded by incisional biopsy, has significantly improved prognosis, with 5-year survival rates exceeding 85% in localized disease (54–56).

#### CT imaging features

**Figure 9:**
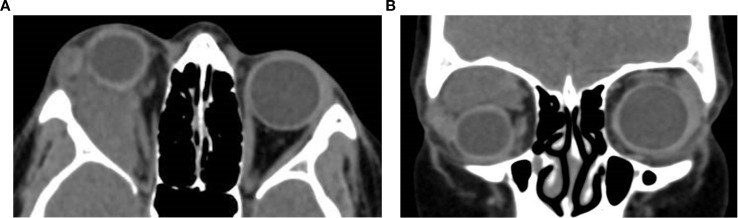
Axial **(a)** and coronal **(b)** contrast-enhanced CT images of an 11-year-old female patient demonstrate a right orbital soft tissue mass that is heterogeneous and poorly circumscribed. The lesion involves the extraconal space and extends medially with moderate displacement of the globe. The mass appears hyperdense relative to orbital fat and is associated with mild proptosis. The lacrimal gland appears separate from the mass with a preserved fat plane. There is no definitive evidence of calcification or bone erosion. It was surgically proven as rhabdomyosarcoma.

Orbital RMS typically appears as an irregular, soft tissue mass with iso- to hyperdense attenuation relative to extraocular muscles. The tumor may involve extraconal, intraconal, or both compartments, with the superonasal quadrant being the most frequent site of origin (54, 57). Enhancement is usually mild to moderate and relatively homogeneous, although heterogeneity may arise from necrosis or hemorrhage. Bone remodeling or erosion is observed in up to 40% of cases, and rare intracranial or sinus extension can occur (53). Calcification is uncommon. The lesion may displace or encase orbital structures such as the globe, optic nerve, or extraocular muscles, but it typically respects the scleral boundary. Notably, the mass lacks encapsulation and often blends with adjacent orbital fat, distinguishing it from well-circumscribed benign tumors. Cavitary RMS, a rare subtype, may appear multilobulated and cystic on CT and MRI, mimicking lymphatic malformations (55) (**Figure 9**).

## Summary

Orbital lesions pose a diagnostic challenge due to their diverse etiologies and overlapping clinical presentations. Accurate distinction among benign, malignant, and inflammatory processes is essential to guide appropriate management, from observation to urgent intervention. CT remains a cornerstone of orbital imaging, offering rapid assessment of lesion location, extent, internal architecture, and bone involvement. This review aims to provide a structured overview of CT features across common orbital pathologies. Important entities outside the scope of this CT-focused review include idiopathic orbital inflammation, IgG4-related orbital disease, neurogenic tumors, secondary spread from eyelids and paranasal sinuses, and orbital metastases. By correlating radiologic findings with clinical characteristics and integrating observations from institutional cases, a practical reference is offered to support diagnostic accuracy and inform clinical decision-making in orbital diseases.
